# Phenformin as prophylaxis and therapy in breast cancer xenografts

**DOI:** 10.1038/bjc.2012.56

**Published:** 2012-02-23

**Authors:** M V C L Appleyard, K E Murray, P J Coates, S Wullschleger, S E Bray, N M Kernohan, S Fleming, D R Alessi, A M Thompson

**Affiliations:** 1 Centre for Oncology and Molecular Medicine, University of Dundee, Dundee DD1 9SY, UK; 2 MRC Protein Phosphorylation Unit, University of Dundee, Dundee DD1 9SY, UK; 3 Division of Pathology and Neuroscience Ninewells Hospital and Medical School, University of Dundee, Dundee DD1 9SY, UK; 4 Department of Surgical Oncology, MD Anderson Cancer Center, 1400 Holcombe Boulevard, Houston, Texas 77030, USA

**Keywords:** breast cancer, phenformin, metformin, xenografts, therapy

## Abstract

**Background:**

Observations that diabetics treated with biguanide drugs have a reduced risk of developing cancer have prompted an enthusiasm for these agents as anti-cancer therapies. We sought to determine the efficacy of the biguanide phenformin in the chemoprophylaxis and in the treatment of oestrogen receptor (ER)-positive MCF7 and receptor triple-negative MDAMB231 xenografts in immunocompromised mice. We also compared the efficacy of phenformin and metformin in the treatment of MDAMB231.

**Methods:**

Immunocompromised mice were divided into groups: (1) phenformin administered for 2 weeks prior to cell injection; (2) established tumours treated with phenformin; (3) established tumours treated with metformin (only for MDAMB231 tumours); (4) untreated controls. Post-treatment tumours, liver and spleen were harvested for further analysis.

**Results:**

Phenformin significantly inhibited both the development and growth of MCF7 and MDAMB231 tumours, and for MDAMB231 at greater efficacy than metformin without murine toxicity. The number of mitotic figures was significantly fewer in xenografts treated with phenformin compared with controls. Results suggested that the mechanism of action of phenformin *in vivo* is consistent with AMPK activation.

**Conclusion:**

Phenformin has clinical potential as an antineoplastic agent and should be considered for clinical trials both in ER-positive and triple-negative breast cancer.

Despite advances in the treatment of and survival from breast cancer, there remains a need to establish additional anti-cancer therapies. Although cancer therapeutics, including those directed against HER2 or PARP, have been developed through intelligent design and other promising agents have emerged from screening assays (Lain *et al*, 2008; Staples *et al*, 2008), the additional and unexpected antineoplastic effect of drugs used in the treatment of non-malignant conditions has been highlighted using epidemiological approaches (Demierre and Nathanson, 2003; Sausville *et al*, 2003). As an exemplar, diabetics treated with biguanide drugs have a reduced risk of developing cancer compared with that of patients receiving sulfonylurea (Evans *et al*, 2005; Landman *et al*, 2010). Metformin is a widely used oral biguanide that originates from the French lilac (*Galega officinalis*), a plant known for several centuries to reduce the symptoms of diabetes mellitus (Chong and Chabner, 2009). Phenformin is a factorially more active drug against tumour cells (Huang *et al*, 2008), but use as an anti-diabetic agent has been limited to relatively few countries (including Italy, Greece, Portugal, Poland, Brazil and Uruguay) because of an increased incidence, compared with metformin, of phenformin-associated lactic acidosis, usually in elderly patients with renal failure (McGuinness and Talbert, 1993).

The biguanides act as oral hypoglycaemic agents primarily through suppression of hepatic glucose production, but also by increased insulin sensitivity, enhanced peripheral glucose uptake, decreased fatty-acid oxidation and decreased absorption of glucose from the gastrointestinal tract (Bailey and Turner, 1996; Kirpichnikov *et al*, 2002).

At the cellular level, biguanides work through activation of AMPK (Zhou *et al*, 2001; Kim *et al*, 2008) in a mechanism that requires LKB1 (Shaw *et al*, 2005; Huang *et al*, 2008) and involves regulation of the downstream pathways relevant to the control of cellular proliferation (Kahn *et al*, 2005), resulting in a variety of effects distinct from their anti-diabetic activity.

In 1968, Lugaro and Giannattasio (1968) described anti-tumour effects of biguanidides. Since then, many other authors have described anti-tumour activity of biguanides in animal models and cell lines. For instance, phenformin has been shown to exert anti-tumour activity in different animal models (Dilman and Anisimov, 1980; Caraci *et al*, 2003) and to enhance the anti-tumour effects of established chemotherapy agents against squamous cell carcinoma, hepatoma-22a and lung cancer in mice (Dilman and Anisimov, 1979). Treatment of the neuroblastoma SH-SY5Y tumour cell line with increasing concentrations of phenformin reduced tumour proliferation and induced apoptosis (Caraci *et al*, 2003). Prophylactic treatment with phenformin was also reported to decrease the incidence of DMBA-induced mammary tumours in rats (Dilman *et al*, 1978).

The potential of biguanides for the prevention and treatment of cancer has focused to date on metformin, widely used in clinical diabetic practice, with clear associations between metformin use in diabetics and the prevention of breast cancer (Evans *et al*, 2005) and increased efficacy of chemotherapy against breast cancer in the neoadjuvant setting (Jiralerspong *et al*, 2009). Indeed, clinical trials using metformin alone or in combination with other therapies in breast cancer are now underway including in the neoadjuvant (Hadad *et al*, 2011) and adjuvant setting (Goodwin *et al*, 2009).

This enthusiasm for metformin has been supported by models of mouse mammary adenocarcinoma in which metformin treatment significantly decreased the incidence and size of the tumours (Anisimov *et al*, 2005) and inhibited the growth of transplantable HER2 mammary carcinoma in mice by 46% (Anisimov *et al*, 2010). There is some controversy regarding efficacy of metformin against triple-negative breast cancer, with reports of growth inhibition of tumour (Liu *et al*, 2009), but also triple-negative breast cancer resistance to metformin (Zhuang and Miskimins, 2008, 2011).

Extending the biguanide class effect beyond metformin, a role for phenformin as a potential anti-cancer agent has recently been demonstrated in a physiological signalling pathway that may inhibit cell growth (Huang *et al*, 2008). Moreover, it has been recently reported that when administered in combination with 2-deoxyglucose, lactic acidosis may be avoided and as phenformin is a more potent drug than metformin, phenformin should be re-examined as a potential agent in cancer therapy (Lea *et al*, 2011).

In the present study, we investigated the efficacy of phenformin in preventing or repressing the growth of tumour xenografts derived from oestrogen receptor (ER)-positive luminal-type MCF7 and receptor triple-negative MDAMB231 breast cancer cell lines and examined the tumour-specific and systemic effects of phenformin on cell proliferation and components of the AMPK and the cell-cycle pathways. We also compared the efficacy of metformin and phenformin against MDAMB231 breast cancer tumour xenografts.

## Materials and methods

### Cell culture and media

MCF7 and MDA-MB-231 cells were acquired from ATCC (LGC Standards, Teddington, UK) and used immediately. Prior to harvesting, cells were grown in DMEM containing 10% fetal calf serum and 1% penicillin/streptomycin at 37 °C with 5% CO_2_ to 80% confluence. Cultured human breast cancer cells were tested for mycoplasma (MycoAlert from Lonza Wokingham Ltd, Slough, UK) prior to harvesting. Mycoplasma-free cells were trypsinised, harvested, rinsed and then suspended in 50 : 50 DMEM and matrigel (BD Biosciences, Two Oak Park, Bedford, MA, USA). The suspension was kept below 4 °C at all times.

### Xenografts

Studies were carried out under licence 60/3729 in accordance with the guidelines of the UKCCCR. Female nude (nu/nu) mice for MCF7 studies or female SCID mice for MDAMB231 studies (both from Harlan, Loughborough, UK) were housed under aseptic conditions in individually ventilated cages in a temperature- (24 °C) and light-controlled (12 h per day) environment, and received autoclaved food and water ad libidum. Nude mice were implanted with 17*β*-estradiol pellets (0.72 mg per pellet) from Innovative Research of America (Sarasota, FL, USA) at least 2 days before injection into each flank of 1 × 10^8^ MCF7 cells in 100 *μ*l of a 50 : 50 DMEM and matrigel suspension. SCID mice were injected subcutaneously in both flanks with 1 × 10^8^ MDAMB231 cells in 100 *μ*l of a 50 : 50 DMEM and matrigel suspension.

### Treatment regime

Mice were randomly divided into groups of 10 mice each and treated as shown in Table 1. In summary, for group 1, 2 weeks prior to injection of MCF7 or MDAMB231, drinking water was replaced with 5% sucrose containing phenformin (300 mg kg^−1^) (pre-treated group). The addition of sucrose was because of palatability. When tumours were ⩾30 mm^3^, water for the mice of the second group was replaced with 5% sucrose containing phenformin (300 mg kg^−1^) and mice of the third group remained untreated as controls, but water was replaced with 5% sucrose. In the case of MDAMB231 tumours, a fourth group had water added with metformin (300 mg kg^−1^). In the case of metformin, palatability was not an issue and the controls in group 5 did not have sucrose added to their water. Drug concentration was based on literature data (Huang *et al*, 2008).

### Tumour measurements

Tumour dimensions and mouse weights were measured twice a week using callipers by one of the authors (MA or KM). Tumour sizes were calculated using the formula *V*=4/3*π*((d1+d2)/4)^3^ mm^3^. Tumour volumes at the end of treatment were compared using Student's *t*-test.

### Tissue processing

When appropriate, animals were killed and tumours were removed, cut in half, each half either fixed in 10% buffered formalin or snap frozen, respectively. Non-tumour tissues (liver and spleen) were snap frozen. Mouse tissues and xenograft tumours were prepared for western blotting or histology and immunohistochemistry analysis as described below.

### Immunohistochemistry

Sections from formalin-fixed paraffin-embedded xenograft tissue (4 *μ*m thick) were cut onto poly-L-lysine-coated glass slides (VWR International Ltd, Leighton Buzzard, UK) and dried for 1 h at 60 °C before being de-paraffinised in Histoclear (National Diagnostics Ltd, Hessle, UK). After blocking endogenous peroxidase activity in 0.5% H_2_O_2_ (100 volumes) in methanol for 35 min at room temperature, antigen retrieval was performed by boiling in 10 mM citric acid buffer, pH 6.0, for 15 min in a microwave oven operating at full power. Sections were allowed to cool, washed in PBS and non-specific antibody-binding blocked in 5% normal goat serum for 30 min at room temperature. Primary rabbit antibodies to phospho-histone H3 (Ser10) or cleaved PARP (Asp 214) (Cell Signaling Technology no. 9701 or no. 9541, respectively, New England Biolabs, Hitchin, UK) were diluted in 5% normal goat serum and incubated overnight at 4 °C. For Ki67 staining, a rat monoclonal anti-Ki67 (Clone TEC-3; DakoCytomation, Dako UK Ltd, Ely, UK) was diluted 1/500 in 5% normal rabbit serum. After washing in PBS, sections were incubated with biotinylated goat-anti-rabbit immunoglobulins diluted 1/250 in 5% normal goat serum or with biotinylated rabbit anti-rat IG (mouse adsorbed, Vector Laboratories Ltd, Peterborough, UK) diluted 1/250 in 5% normal rabbit serum, washed in PBS and incubated with avidin–biotinylated peroxidase complex in PBS using Vectastain Elite ABC kit reagents (Vector Labs). Immunoreactive sites were identified with diaminobenzidine/H_2_O_2_ in PBS containing 5 mM imidazole, pH 7.0, for 10 min and sections counterstained with Mayer's haematoxylin before dehydrating, mounted with coverslips using DPX and viewed using light microscopy.

### Histology

The morphology of the tumours was assessed by haematoxylin- and eosin-stained sections. To assess changes in collagen content in treated tumours, Van Gieson staining was performed. Because formalin-fixed tissues can be significantly affected by differences in fixation time rendering TUNEL assays unreliable in many cases, to assess apoptosis and mitosis in tumour tissues, sections from formalin-fixed paraffin-embedded xenograft tissue were stained with Hematoxylin and Eosin. Cells in 20 microscopic fields were counted and the percentage of mitotic and apoptotic cells determined. Populations were compared using a two-tailed, two-sample equal variance Student's *t*-test.

### Western blots

To confirm the activation of the AMPK pathway by phenformin *in vivo* in these murine models, AMPK, phospho-AMPK, was examined in the liver, spleen and tumours of treated mice. Tissues were homogenised on ice in a 10-fold mass excess of ice-cold lysis buffer comprising 50 mM Tris-HCl, pH 7.5, 1 mM EGTA, 1% (w/v) Triton-X 100, 1 mM sodium orthovanadate, 50 mM sodium fluoride, 5 mM sodium pyrophosphate, 0.27 M sucrose, 0.1% (v/v) 2-mercaptoethanol and ‘Complete’ protease inhibitor cocktail (Roche Diagnostics Ltd, Burgess Hill, UK) using a Kinematica Polytron homogeniser (Kinematica Inc., Bohemia, NY, USA). Tissue lysates were centrifuged at 18 000 **g** for 15 min at 4 °C and the supernatant was snap frozen and stored at −80 °C. The protein concentration was determined by Bradford assay (Pierce-Fisher Scientific Ltd, Loughborough, UK). Total tissue lysate 20 *μ*g was heated at 70 °C for 5 min in SDS–PAGE sample buffer (50 mM Tris-HCl, pH6.8, 6.5% (v/v) glycerol, 1% (w/v) SDS and 1% (v/v) 2-mercaptoethanol), subjected to PAGE and electrotransferred to nitrocellular membranes. Membranes were blocked for 1 h in TBS-Tween (50 mM Tris-HCl, pH 7.5, 0.15 M NaCl and 0.1% Tween 20) containing 10% (w/v) skimmed milk powder and probed with the indicated antibodies in TBS-Tween containing 5% (w/v) BSA for 16 h at 4 °C. Detection was performed using horseradish peroxidase-conjugated secondary antibodies (Pierce) and ECL reagent. Anti-AMPK*α*1, raised in sheep against the peptide CTSPPDSFLDDHHLTR (residues 344–358 of rat AMPK*α*1), and AMPK*α* p-T172 (no. 2535) were purchased from Cell Signaling Technology.

## Results

### Efficacy of phenformin in an MCF7 xenograft model

**Figure 1:**
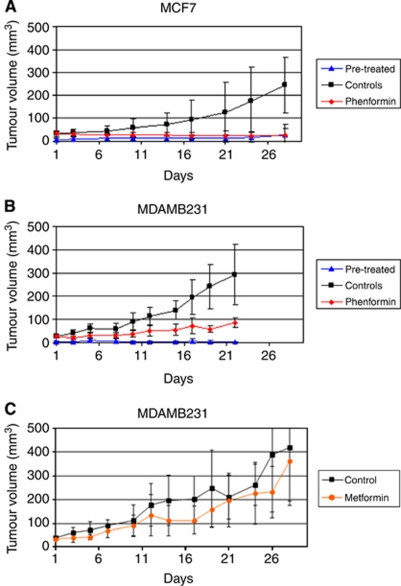
Effect of 300 mg kg^−1^ phenformin on human breast MCF7 (**A**) and MDAMB231 (**B**), and 300 mg kg^−1^ metformin on MDAMB231 (**C**) tumour xenografts. For the phenformin experiment, mice were divided into three groups. Controls received no phenformin. Pre-treatment mice were given phenformin (300 mg kg^−1^) in 5% sucrose instead of normal drinking water for 2 weeks prior to injection of MCF7 or MDAMB231 cells. The phenformin group received normal drinking water until tumours reached ⩾30 mm^3^, after which drinking water was replaced with 5% sucrose containing phenformin (300 mg kg^−1^). Control group also had water replaced with 5% sucrose. For the metformin experiment, mice were divided into two groups. Controls received no metformin and mice with established tumours received metformin (300 mg kg^−1^) in water. MCF7 tumours pre-treated or treated with phenformin had statistically significant inhibition of tumour growth of 88% relative to the control group (*P*<0.05). Animals injected with MDAMB231 cells and treated prophylactically with phenformin showed small lumps 6 weeks after inoculation, which remained static for the rest of the experiment. Established MDAMB231 tumours treated with phenformin demonstrated statistically significant inhibition of tumour growth of 60% relative to the control group (*P*<0.05). There were no statistically significant differences between control mice and mice treated with metformin.

Mice pre-treated with phenformin had delayed establishment of tumours that were fewer in number: after 4 weeks, 25% of mice developed tumours that reached a mean 24 mm^3^. In contrast, without phenformin pre-treatment, 3 weeks after inoculation, 60% of untreated mice had tumours. Established MCF7 tumours treated with phenformin had 88% inhibition of tumour growth relative to the control group (Student's *t*-test *P*<0.05) (Figure 1A).

### Efficacy of phenformin in an MDAMB231 xenograft model

In the MDAMB231 xenograft model, 60% of the untreated animals had measurable tumours 5 weeks after inoculation, whereas mice treated prophylactically with phenformin showed small, static lumps 6 weeks after inoculation. Established MDAMB231 tumours treated with phenformin demonstrated statistically significant inhibition of tumour growth of 60% relative to the control group (Student's *t*-test *P*<0.05) (Figure 1B).

### Efficacy of metformin in an MDAMB231 xenograft model

Established MDAMB231 tumours treated with metformin did not show statistically significant inhibition of tumour growth compared with the control group (Figure 1C).

### Histology

Histological examination confirmed that the tumours consisted of high-grade adenocarcinomas. Hematoxylin- and Eosin-stained MCF7 control tumours showed 1.4±0.8% apoptotic cells and 1.4±0.8% mitotic cells. In tumours treated with phenformin, the percentage of apoptotic cells was 1.05±0.6%, not significantly different to control tumours (*P*>0.05). However, the number of mitotic cells significantly decreased in treated tumours compared with untreated controls (0.18±0.2%, *P*<0.05, Student's *t*-test). The same pattern was observed for MDAMB231 tumours, where the number of apoptotic cells remained unchanged upon treatment with phenformin (0.7±0.38% for control tumours and 0.61±0.29% for phenformin-treated tumours, *P*>0.05, Student's *t*-test), but there was a significant decrease in the proportion of mitotic cells in phenformin-treated tumours (0.4±0.24%) compared with controls (0.9±0.46%) (*P*<0.005, Student's *t*-test).

**Figure 2:**
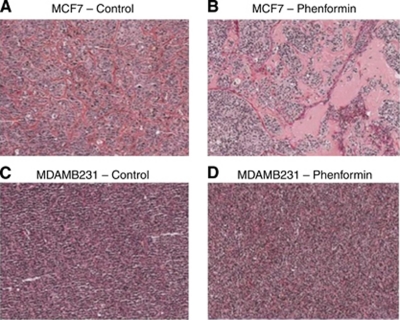
Connective tissue histological analysis of MCF7 and MDAMB231 xenografts treated with phenformin using Van Gieson stain. MCF7 tumours treated with phenformin showed substantial increase of connective tissue, demonstrated as red/pink staining, which replaces epithelial tumour cells (**A** and **B**). No differences were observed in MDAMB231 tumours (**C** and **D**), × 5 magnified image captured with an Aperio ScanScope XT, Aperio Technologies, Vista, CA, USA.

In MCF7 tumours treated with phenformin, there was an increase in stromal connective tissue but not proliferation compared with control tumours, observed with Van Gieson staining used to differentiate between collagen and smooth muscle (Figures 2A and B). MDAMB231 tumours, however, did not show differences in the amount of stromal connective tissue between phenformin-treated and control tumours (Figures 2C and D).

### Immunohistochemistry

**Figure 3:**
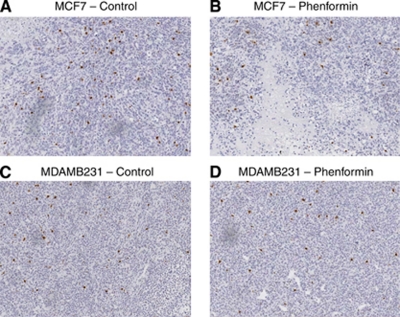
Phospho-histone H3 immunohistochemistry analysis of MCF7 and MDAMB231 xenografts treated with phenformin. Tumours were harvested and immunohistochemistry analysis performed as described in Materials and methods. No significant differences were observed for phenformin (**B** and **D**) compared with untreated MCF7 and MDAMB231 tumours (**A** and **C**), × 5 magnified image captured with an Aperio ScanScope XT, Aperio Technologies.

**Figure 4:**
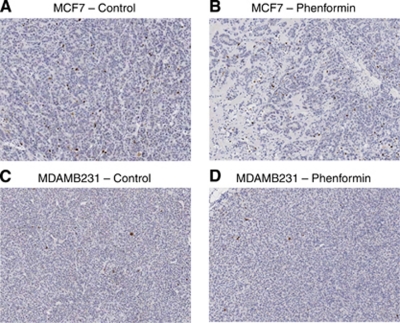
Cleaved PARP analysis of MCF7 and MDAMB231 xenografts treated with phenformin. Tumours were harvested and immunohistochemistry analysis performed as described in Materials and methods. No significant differences were observed for phenformin (**B** and **D**) compared with untreated MCF7 and MDAMB231 tumours (**A** and **C**), × 5 magnified image captured with an Aperio ScanScope XT, Aperio Technologies.

**Figure 5:**
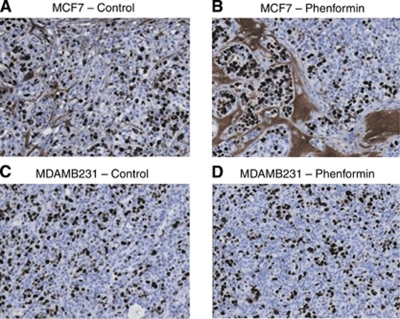
Ki67 immunohistochemistry analysis of MCF7 and MDAMB231 xenografts treated with phenformin. Tumours were harvested and immunohistochemistry analysis performed as described in Materials and methods. No significant differences were observed for MCF7 tumour treated with phenformin compared with control (**A** and **B**). Similar results were found for MDAMB231 tumours (**C** and **D**), × 5 magnified image captured with an Aperio ScanScope XT, Aperio Technologies.

No significant differences were observed in immunohistochemical detection of phospho-histone H3, a well-established marker for mitosis (Figure 3), cleaved PARP, a marker for apoptosis (Figure 4), or the proliferation marker Ki67 (Figure 5) between control tumours and the two therapeutic regimes: tumours from phenformin pre-treated mice and established tumours treated with phenformin.

### AMPK activation analysed by western blot

**Figure 6:**
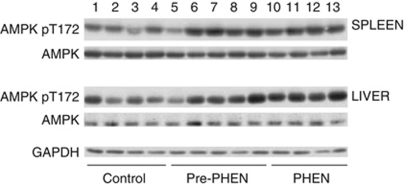
AMPK activation in liver and spleen of mice. Liver and spleen of control mice and mice that were pre-treated or treated after xenograft establishment with 300 mg kg^−1^ phenformin were processed and western blots produced as described in Materials and methods. Lanes 1–4 show the results for control mice, lanes 5–9 show the results for mice pre-treated with phenformin and lanes 10–13 show the results for mice treated with phenformin. Phosphorylation of the activation loop of AMPK (T172) was enhanced in liver and spleen from mice pre-treated or treated (lanes 10–13) with phenformin compared with untreated control mice.

**Figure 7:**
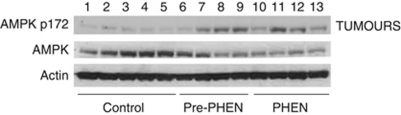
AMPK activation in MDAMB231 xenograft tumours. Tumours of control mice and mice that were pre-treated or treated with 300 mg kg^−1^ phenformin were processed and western blots produced as described in Materials and methods. Lanes 1–5 show the results for control mice, lanes 6–9 for mice pre-treated with phenformin and lanes 10–13 for mice treated with phenformin. Phosphorylation of the activation loop of AMPK (T172) was enhanced in most of the tumours from mice pre-treated or treated with phenformin compared with untreated control mice.

Phosphorylation of the activation loop of AMPK (T172) was enhanced in liver, spleen (Figure 6) and tumour lysates (Figure 7) derived from mice administered with phenformin compared with untreated control mice.

## Discussion

This study has demonstrated the efficacy of phenformin *in vivo* in the prevention and growth reduction of ER-positive, luminal A, MCF7 and triple-negative MDAMB231 xenograft tumours in immunocompromised mice. Phenformin demonstrated greater inhibition of MDAMB231 tumour growth than metformin in treated animals. Resistance to metformin by MDAMB231 has been reported by other authors (Zhuang and Miskimins, 2011). The fact that phenformin treatment may lead to lactic acidosis whereas metformin does not suggests that these biguanides work through the pathways that are not identical, which may account for the difference in sensitivity by MDAMB231.

There was a significant decrease in the number of mitotic figures in treated tumours compared with untreated controls for both cell line-derived xenograft models withno change in the number of apoptotic cells, suggesting cell-cycle arrest. This is consistent with AMPK-induced cell-cycle arrest in hepatoma HepG2 cells (Imamura *et al*, 2001), human aortic smooth muscle cells, rabbit aortic strip (Igata *et al*, 2005) and mouse embryonic fibroblasts (Jones *et al*, 2005).

MCF7, but not MDAMB231, tumours treated with phenformin showed a substantial increase in stromal connective tissue (Figures 3A and B) compatible with effects of the drug on activated fibroblasts (Olumi *et al*, 1999; Bhowmick and Moses, 2005). The increased stroma associated with a decrease in mitotic figure numbers observed for MCF7 tumours treated with phenformin is consistent with a stroma not supportive of tumour growth. Although stroma is usually considered to be associated with invasion and metastasis, there have been reports of stroma formation that inhibits the growth of several cancers in a mode of action that may be due to the formation of tumour stroma not supportive of tumour growth (Skobe *et al*, 1997; Meyerhardt and Mayer, 2005; Chlenski *et al*, 2006, 2007). Moreover, our results for cleaved PARP or phospho-histone H3 immunohistochemistry with no increase in pre-treated/treated tumours compared with controls are supportive of the cell-cycle arrest hypothesis. Cell proliferation, measured by Ki67, was not significantly lower in either of the phenformin-treated tumour groups compared with controls (Figure 5). However, there was a significant decrease in the number of mitotic figures in treated tumours compared with untreated controls for both cell lines with no change in the number of apoptotic cells, suggesting cell-cycle arrest. This underlines that Ki67 fraction and mitotic activity index are not necessarily interchangeable in these breast cancer xenograft models and may represent differences in the kinetics of cell-cycle progression (Rudolph *et al*, 1998; Jalava *et al*, 2006).

AMPK activation was observed by western blots using phospho-specific antibodies in the liver, spleen and tumours of mice treated or pre-treated with phenformin, consistent with a proposed mechanism of action of biguanides involving AMPK activation leading to mTORC1 inhibition (Zhang *et al*, 2007; Hadad *et al*, 2008).

## Conclusion

Phenformin significantly inhibited both the development and growth of established MCF7 and MDAMB231 tumours without murine toxicity. The potential for phenformin should be considered further as an antineoplastic agent of greater *in vivo* efficacy than metformin for the treatment of breast cancer.

## Figures and Tables

**Table 1:** Treatment regime for MCF7 and MDAMB231 xenografts using phenformin and metformin

**Cell line**	**MCF7**	**MDAMB231**
Group 1 (pre-treated)	Phenformin in 5% sucrose added 2 weeks prior to cell injection	Phenformin in 5% sucrose added 2 weeks prior to cell injection
Group 2	Phenformin in 5% sucrose added after tumour development	Phenformin in 5% sucrose added after tumour development
Group 3	Untreated control (water replaced with 5% sucrose)	Untreated control (water replaced with 5% sucrose)
Group 4	—	Metformin in drinking water added after tumour development
Group 5	—	Untreated control
